# Non-proctology

**Published:** 1917-12

**Authors:** Jerome Morley Lynch

**Affiliations:** New York City


### NON-PROCTOLOGY. A GLIMPSE INTO THE FUTURE.

By Jerome Morley Lynch, M.D., F.A.C.S., New York City.

We cannot claim for proctology that it is a new field of effort. It was practiced centuries ago. In early records are described the cutting of a fistula and the ligature operation for hemorrhoids. While, therefore, technic offers no novelties, physiologic surgery affords a most promising field for the further development of Proctology.

The observations of the ancients were remarkably accurate, and, considering the limited amount of detailed knowledge at their command, are as wonderful examples of the power of human induction as many of the best conclusions of the modern day. Consider, for example, their decision that a hemorrhoidal flux was in some cases to be regarded as beneficient. Whether or not the cause was known, as we understand it to-day, to be hepatic cirrhosis or right heart engorgement, still, for all, the observation is based upon as sound philosophy as could possibly be adduced to-day.

It is well for us, in the occasional mental stock-taking (which is periodically forced upon us) to bear in mind that the tendency of modern scientific instruments of precision, together with all the wonderful array of laboratory tests, while of indisputable value, are in a certain sense of greater value to the patient than to the physician; for they certainly tend to take from the latter the necessity for that keenness of perception and correlative interpretation of symptoms, which was the distinguishing characteristic of the earlier physicians whose chief reliance was their own mental activity.

